# CTLA-4 Antisense Oligonucleotide Contributes to Enhanced Immunogenicity of an Adjuvanted Recombinant *Sporothrix* spp. Enolase Antigen

**DOI:** 10.3390/vaccines14040334

**Published:** 2026-04-09

**Authors:** Giovanna Justino Momente, Deivys Leandro Portuondo, Adriana Fernandes de Deus, Matheus Ricardo Curti Gonçalves, Fernanda Luiza Piccineli, Tarcila Pavicic Catalan de Oliveira Campos, Damiana Téllez-Martínez, Iracilda Zeppone Carlos, Alexander Batista-Duharte

**Affiliations:** 1 Department of Clinical Analysis, School of Pharmaceutical Sciences, São Paulo State University (UNESP), Araraquara 14800-903, SP, Brazil; giovanna.j.momente@unesp.br (G.J.M.); deivysleandro@gmail.com (D.L.P.); adriana.deus@unesp.br (A.F.d.D.); mcurtivet@gmail.com (M.R.C.G.); fernanda.piccineli@unesp.br (F.L.P.); tarcila.catalan@unesp.br (T.P.C.d.O.C.); damiana.tellez-martinez@unesp.br (D.T.-M.); 2 Department of Cell Biology, Physiology and Immunology, University of Cordoba, Av. Menendez Pidal s/n, 14004 Cordoba, Spain; 3 Immunology and Allergy Group (GC01), Maimonides Biomedical Research Institute of Cordoba (IMIBIC), Reina Sofia University Hospital, 14004 Cordoba, Spain

**Keywords:** sporotrichosis, CTLA-4, antisense oligonucleotide, enolase vaccine, immune checkpoint modulation, antifungal immunotherapy

## Abstract

**Background/Objectives**: Sporotrichosis is an emerging zoonotic subcutaneous fungal infection with limited therapeutic options, highlighting the need for improved immunomodulatory strategies. CTLA-4 is an inhibitory immune checkpoint that negatively regulates T-cell activation. In this study, we evaluated whether a CTLA-4 antisense oligonucleotide (CTLA-4 ASO) is associated with enhanced immune responses to an adjuvanted recombinant *Sporothrix* sp. enolase antigen (rSsEno) formulation. **Methods**: CTLA-4 ASO uptake, cytotoxicity, and gene-silencing activity were assessed in murine splenocytes in vitro. BALB/c mice were immunized with rSsEno formulated with Montanide Gel 01, either alone or in combination with 5 µg CTLA-4 ASO. Antigen-specific serum antibody responses were quantified by ELISA. Splenocytes from immunized mice were restimulated with enolase, and cytokine production (IFN-γ, IL-2, IL-17, and TNF-α) was measured using Cytometric Bead Array (CBA). **Results**: CTLA-4 ASO was efficiently internalized by splenocytes and was associated with reduced expression of CTLA-4 without detectable cytotoxicity in vitro. Mice receiving the ASO-supplemented formulation developed significantly higher anti-enolase antibody titers compared to those immunized with adjuvant alone. Upon antigen restimulation, splenocytes from ASO-treated mice produced higher levels of IFN-γ, IL-2, TNF-α, and IL-17, consistent with an enhanced recall response characterized by a mixed Th1/Th17 cytokine profile. **Conclusions**: CTLA-4 ASO was associated with an enhanced recall response characterized by a mixed Th1/Th17 cytokine profile. These findings suggest a potential immunomodulatory effect of CTLA-4 targeting. Further studies incorporating dose optimization, infection challenge models, and appropriate sequence controls are required to determine the specificity and relevance of these effects for protective immunity against sporotrichosis.

## 1. Introduction

Cytotoxic T-lymphocyte-associated protein 4 (CTLA-4) is a key inhibitory receptor that regulates T-cell activation and maintains immune homeostasis. It functions as a central inhibitory “checkpoint” that dampens T-cell priming by competing with CD28 for CD80/CD86 and by modulating antigen-presenting cell costimulation. CTLA-4 is also constitutively expressed by regulatory T cells (Tregs), where it contributes to dominant immune suppression [1]. In this setting, CTLA-4 functions as a physiological brake on adaptive immunity. Although critical for maintaining immune tolerance and preventing immunopathology [2,3], its activity can also limit the development of protective responses, supporting strategies aimed at checkpoint modulation [4].

In recent years, molecular adjuvants that modulate immunoregulatory pathways have been explored to improve vaccine immunogenicity and protective efficacy. Approaches that transiently suppress inhibitory networks, including those linked to regulatory T cells, have gained attention [4]. Gene-targeting platforms directed at immune checkpoints and other immunosuppressive pathways represent a growing strategy in this field [5]. CTLA-4 blockade has shown immunomodulatory value in vaccine design, with evidence that its inhibition enhances antigen-specific immune responses [6,7,8,9,10,11,12,13]. Greater efficacy is observed when vaccines are combined with co-stimulatory strategies that lower T-cell activation thresholds or counter inhibitory signals [5]. Consistently, co-administration of vaccines with anti–CTLA-4 monoclonal antibodies improve antitumor responses compared with either treatment alone [1,6], and has been proposed as a potential adjunct in chronic infections when paired with therapeutic vaccines [12,13].

Previous murine studies using FOXP3 gene-silencing have shown that transient attenuation of regulatory T-cell activity enhances immune responses to vaccination and infection, leading to stronger antigen-specific cellular and humoral immunity and improved pathogen control [14,15]. These findings support modulation of immunosuppressive pathways as an immunopotentiation strategy. However, FOXP3-targeted approaches are largely restricted to the Treg compartment, whereas immune regulation also depends on checkpoint receptors expressed across multiple T-cell subsets and activation stages. Accordingly, immune checkpoint pathways are being explored as complementary or alternative immunomodulatory targets. CTLA-4 targeting may provide broader control of inhibitory signaling during antigen priming and effector phases, with potential to enhance antigen-specific immune responses beyond Treg-centered strategies [12,13]. Such approaches may be especially valuable for vaccines against difficult-to-treat fungal infections.

Sporotrichosis is an expanding zoonotic mycosis in Brazil, mainly caused by *Sporothrix brasiliensis* and largely transmitted from cats to humans, driving urban spread and growing public health impact [16,17]. This species is more virulent than other members of the genus and is linked to higher fungal burden and more severe disease [18,19,20]. Infection is often difficult to cure, requires prolonged antifungal therapy, and shows poorer outcomes in immunocompromised patients [21,22,23]. The extended and costly treatment adds a significant socioeconomic burden, particularly in low-resource settings [24]. In this study, we evaluated whether incorporating a low dose of a CTLA-4 antisense oligonucleotide (CTLA-4 ASO) into the formulation could enhance immunogenicity in a model established in our laboratory using a vaccine candidate based on recombinant *Sporothrix* sp. enolase antigen (rSsEno) as a proof of concept [25]. We analyzed the uptake kinetics of CTLA-4 ASO in CD4+ T cells and assessed its effects on antigen-specific immune responses, including antibody production and Th1/Th17-associated cytokine responses. We also evaluated its cytotoxicity and its ability to modulate the immune response elicited by the vaccine formulation. Overall, this work provides proof-of-concept evidence for the immunomodulatory potential of CTLA-4 silencing in this context and establishes a basis for more in-depth mechanistic and functional studies.

## 2. Materials and Methods

### 2.1. Oligonucleotides (ONs)

To evaluate CTLA-4 interference, antisense oligonucleotide (ASO) sequences were designed against the mouse Ctla4 transcript. The sequences used were as follows:

CTLA-4 ASO: 5′-GmAmAGAGTGAGCAmGmGmG-3′ (15 bp);

scrambled control: 5′-mAmGmGAGGACAGGAGAGmAmG*mA-3′ (18 bp).

**Figure A1:**
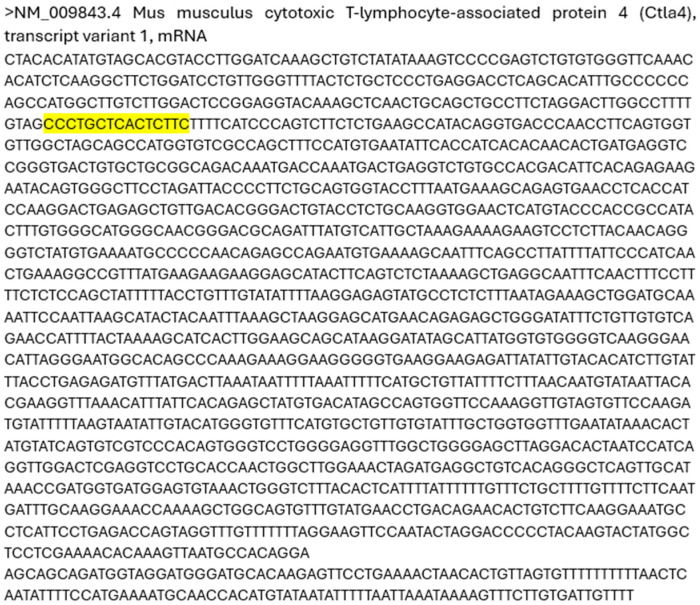
Mus musculus cytotoxic T-lymphocyte-associated protein 4 (Ctla4), transcript variant 1, mRNA NCBI Reference Sequence: NM_009843.4. Yellow: Region complementary to the CTLA-4 ASO.

The CTLA-4 ASO sequence is complementary to a region located within exon 1 of the mouse Ctla4 gene (Mus musculus; chromosome 1, GRCm39 assembly; NCBI Reference Sequence NC_000067.7). The targeted sequence corresponds to the Ctla4 transcript variant 1 mRNA (NCBI RefSeq: NM_009843) (Figure A1). The CTLA-4 ASO and the scrambled control oligonucleotide were used as previously described [14]. All oligonucleotides were obtained from Integrated DNA Technologies (IDT, Coralville, IA, USA).

### 2.2. Animals

Six-week-old male BALB/c mice were obtained from the PRPI-USP Animal Facility Network, Ribeirão Preto Campus (USP). Animals were housed in mini-isolators under controlled environmental conditions (23 ± 2 °C and 56% relative humidity) under a 12 h light/dark cycle, with food and water available ad libitum. All experimental procedures were approved by the Institutional Ethics Committee for Animal Research of the School of Pharmaceutical Sciences of Araraquara–UNESP (Protocol CEUA/FCF/CAr: 10/2023), approved on 10 July 2023.

### 2.3. Immunization Scheme

**Figure 1:**
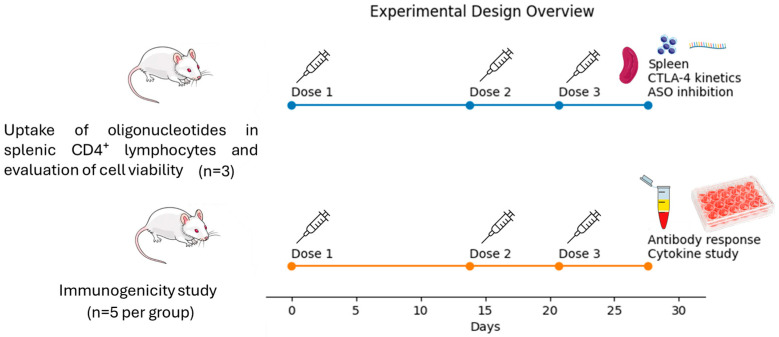
Experimental design overview. Two independent experimental approaches were performed. The first study (n = 3) was designed for in vitro assays, including evaluation of CTLA-4 expression kinetics, ASO inhibitory activity, oligonucleotide uptake, and cell viability. The second study (n = 5 per group) was conducted to assess vaccine immunogenicity. Mice were immunized subcutaneously on days 0, 14, and 21 with 100 μg of rSsEno formulated with 10% Gel 01 in a final volume of 100 μL. For the immunogenicity study, mice were assigned to the following groups: (1) PBS, (2) rSsEno (100 μg/100 μL), (3) Gel 01 (10%) + rSsEno (100 μg/100 μL), and (4) Gel 01 (10%) + rSsEno (100 μg/100 μL) + CTLA-4 ASO (5 μg/100 μL). CTLA-4 ASO was administered at 5 μg per mouse based on previously reported studies. Seven days after the final immunization, mice were euthanized by CO_2_ inhalation, and spleens were aseptically collected for downstream immunological analyses.

To determine the time of maximal CTLA-4 expression, mice (n = 3) received three subcutaneous doses of recombinant *Sporothrix* sp. enolase (rSsEno; 100 µg/100 µL) formulated with Gel 01 (10%). Splenocytes were subsequently harvested and stimulated in vitro with rSsEno (10 µg/mL) for CTLA-4 expression analysis. For in vitro evaluation of CTLA-4 ASO inhibitory activity, mice (n = 3) were immunized following the same regimen. Splenocytes were cultured under the following conditions: RPMI 1640 culture medium (Sigma-Aldrich, St. Louis, MO, USA) alone, rSsEno (10 µg/mL), or rSsEno (10 µg/mL) in combination with CTLA-4 ASO (5 µg/100 µL). For the immunogenicity study, mice (n = 20) were randomly assigned to experimental groups and immunized with three doses of the following formulations: PBS (Phosphate-Buffered Saline); rSsEno (100 µg/100 µL); Gel 01 (10%) + rSsEno (100 µg/100 µL); or Gel 01 (10%) + rSsEno (100 µg/100 µL) + CTLA-4 ASO (5 µg/100 µL). For cytometric analyses, splenocytes were stimulated in vitro with rSsEno (10 µg/mL) (Figure 1).

### 2.4. Uptake of Oligonucleotides in Splenic CD4^+^ Lymphocytes

Spleens from three mice were aseptically removed, and splenocyte suspensions were prepared as previously described [15]. Two Cy5-labeled oligonucleotides (ONs) were evaluated: a CTLA-4 ASO and a scrambled control ON. An untreated (vehicle-only) condition was included as a control. Splenocytes were incubated with each ON at a final concentration of 5 µM for 1 h at 37 °C in RPMI 1640 culture medium containing 10% fetal bovine serum (FBS, Gibco, Thermo Fisher Scientific, Waltham, MA, USA).

### 2.5. Cellular Viability

The cytotoxicity of rSsEno, the CTLA-4 ASO, and its scrambled control was evaluated individually or in combination at the same concentrations used in the functional assays. Cells were treated under the indicated conditions for 48 h. After incubation, cell viability and apoptosis were assessed by Annexin V–FITC (BD Biosciences, San Jose, CA, USA) and Propidium Iodide (PI) (Sigma-Aldrich, St. Louis, MO, USA). Briefly, cells were harvested, washed with PBS, resuspended in buffer, and stained with Annexin V–FITC and PI. Samples were incubated in the dark at room temperature and subsequently analyzed by flow cytometry. Cell populations were defined as viable (PI^−^ Annexin V^−^ and PI^−^ Annexin V^+^), early apoptotic (PI^−^ Annexin V^+^), late apoptotic (PI^+^ Annexin V^+^), or necrotic (PI^+^ Annexin V^−^).

### 2.6. Determination of Peak CTLA-4 Expression

To establish the optimal time point for CTLA-4 ASO administration, the kinetics of CTLA-4 expression in CD4^+^ T cells were evaluated following antigen-specific stimulation. Single-cell suspensions were obtained by mechanically disrupting spleens in RPMI 1640 medium supplemented with 5% heat-inactivated FBS, 100 U/mL penicillin (Sigma-Aldrich, St. Louis, MO, USA), 100 μg/mL streptomycin (Sigma-Aldrich, St. Louis, MO, USA), and 2 × 10^−5^ M 2-mercaptoethanol (Sigma-Aldrich, St. Louis, MO, USA). Residual erythrocytes were eliminated using ammonium chloride lysis buffer. Cells were washed and viability was assessed by trypan blue exclusion. Splenocytes were resuspended at a concentration of 1 × 10^6^ cells/mL in PBS supplemented with 1% bovine serum albumin (BSA, Sigma-Aldrich, St. Louis, MO, USA). 

Cells were maintained in complete RPMI 1640 medium with rSsEno (10 μg/mL) for 0, 24, 48, 72, and 96 h at 37 °C in 5% CO_2_. At each time point, cells were incubated with anti-mouse CD16/CD32 Fc Block (clone 2.4G2, BD Biosciences, San Jose, CA, USA) for 20 min at 4 °C. Surface staining was performed using anti-CD3-FITC (clone 17A2, BD Biosciences, San Jose, CA, USA), anti-CD4-APC (clone RM4-5, BD Biosciences, San Jose, CA, USA), and anti-CTLA-4-PE (clone UC10-4F10-11, BD Biosciences, San Jose, CA, USA) for 30 min at 4 °C in the dark. After washing PBS containing 1% BSA, cells were resuspended in acquisition buffer. Data were acquired using a BD Accuri™ C6 flow cytometer (BD Biosciences, San Jose, CA, USA) and analyzed with FlowJo software, version 10.6.2 (Tree Star Inc., Ashland, OR, USA). Lymphocytes were identified based on forward and side scatter parameters (FSC/SSC), followed by gating on CD3^+^CD4^+^ T cells. CTLA-4 expression was determined as the percentage of CTLA-4^+^ cells within the CD3^+^CD4^+^ population. A minimum of 50,000 events were collected per sample.

### 2.7. In Vitro Evaluation of CTLA-4 ASO Inhibitory Activity

After establishing that CTLA-4 expression peaked at 48 h, the inhibitory effect of the CTLA-4 ASO was evaluated under in vitro conditions. Splenocytes were aseptically harvested and single-cell suspensions were prepared and adjusted to 1 × 10^6^ cells/mL in complete RPMI 1640 medium. Cells were cultured for 48 h at 37 °C in 5% CO_2_ under the following conditions (performed in duplicate): (i) RPMI 1640 medium alone (ii) rSsEno (10 μg/mL), and (iii) rSsEno (10 μg/mL) in combination with CTLA-4 ASO (5 μg/100 μL).

Following incubation, cells were incubated with anti-mouse CD16/CD32 Fc Block (clone 2.4G2, BD Biosciences, San Jose, CA, USA) for 20 min at 4 °C. Surface staining was then performed using anti-CD3-FITC (clone 17A2), anti-CD4-APC (clone RM4-5), and anti-CTLA-4-PE (clone UC10-4F10-11) (all from BD Biosciences, San Jose, CA, USA) for 30 min at 4 °C in the dark. After washing with PBS containing 1% BSA, samples were acquired on a BD Accuri™ C6 flow cytometer and analyzed using FlowJo software. CTLA-4 expression was quantified as the percentage of CTLA-4^+^ cells within the gated CD3^+^CD4^+^ T cell population. The reduction in CTLA-4 expression in ASO-treated cultures relative to antigen-stimulated controls was used to determine the inhibitory efficacy of the ASO.

### 2.8. Immunogenicity Study in BALB/c Mice

The vaccine formulation consisted of rSsEno combined with 10% Gel 01 adjuvant (Seppic, France), prepared according to the manufacturer’s instructions and as previously described [25]. To assess the impact of CTLA-4 silencing on the immune response, CTLA-4 ASO was co-administered with the vaccine in one experimental group. PBS was used as a negative control, as described in Section 2.3.

#### 2.8.1. Antigen-Specific IgG and IgG2b Antibody Responses

Antigen-specific IgG and IgG2b antibody levels were determined by enzyme-linked immunosorbent assay (ELISA). Briefly, 96-well flat-bottom microplates (Corning, New York, NY, USA) were coated with rSsEno at 20 μg/mL (100 μL/well) diluted in carbonate–bicarbonate coating buffer (0.5 M, pH 9.6) and incubated overnight at 4 °C. Plates were washed with PBS containing 0.1% Tween 20 (PBS-T) and blocked with 200 μL/well of 2% BSA in PBS-T for 1 h at room temperature. After washing, individual serum samples from mice immunized with PBS, rSsEno, Gel 01 + rSsEno, or Gel 01 + rSsEno + CTLA-4 ASO were diluted 1:80 in PBS-T and added to the plates in duplicate. Plates were incubated for 1.5 h at 37 °C and washed with PBS-T. Horseradish peroxidase (HRP)-conjugated goat anti-mouse IgG or IgG2b secondary antibodies were added and incubated for 1 h at 37 °C. Following washing, 3,3′,5,5′-tetramethylbenzidine (TMB) substrate solution (Sigma-Aldrich, St. Louis, MO, USA) was added for color development. The reaction was stopped with 50 μL of 1 N H_2_SO_4_, and optical density (OD) was measured at 450 nm using a Multiskan Ascent microplate reader (Labsystems Research, Helsinki, Finland).

#### 2.8.2. Cytokine Quantification

Seven days after the last immunization, spleens were aseptically collected and single-cell suspensions were prepared as described above. Splenocytes (1 × 10^6^ cells/mL) were cultured in complete RPMI 1640 medium and stimulated ex vivo with rSsEno (10 μg/mL) for 48 h at 37 °C in 5% CO_2_. Culture supernatants were collected and stored at −80 °C until analysis. Cytokine concentrations (IFN-γ, IL-17, IL-2, and TNF-α) were quantified using CBA kit (BD Biosciences, San Jose, CA, USA) according to the manufacturer’s instructions and analyzed by flow cytometry.

### 2.9. Statistical Analyses

Statistical analyses were conducted using Prism software version 6.01 (GraphPad, San Diego, CA, USA). Differences among groups were evaluated by one-way analysis of variance (ANOVA) followed by Tukey’s multiple comparison test. A 95% confidence level was applied in all analyses. Statistical significance was defined as * (*p* < 0.05); ** (*p* < 0.01); *** (*p* < 0.001); **** (*p* < 0.0001).

## 3. Results

### 3.1. Primary Sequence of the CTLA-4 Gene in FASTA Format and Target Region for CTLA-4 Silencing

According to the most recent NCBI annotation (Figure A1), the antisense ASO used to suppress CTLA-4 expression targets a sequence within Exon 1, allowing hybridization to both pre-mRNA and mature mRNA transcripts. Based on this positioning, gene silencing may result from RNase H-mediated cleavage of the ASO–RNA duplex or, if overlapping splicing regulatory elements, from steric blockade interfering with splice-site recognition and normal splicing processes.

### 3.2. Uptake of Oligonucleotides in Splenic CD4^+^ Lymphocytes and Cell Viability

**Figure 2:**
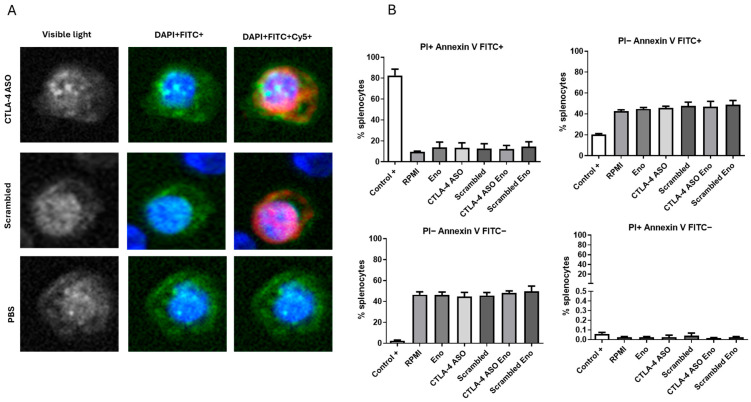
Uptake of oligonucleotides in splenic CD4^+^ lymphocytes and cell viability assessment. (**A**) Splenocytes were incubated for 1 h with 5 µM Cy5-labeled CTLA-4 ASO, Cy5-labeled scrambled control, or vehicle (PBS). Cells were then stained with anti-mouse CD4–FITC, fixed with 4% formaldehyde, and analyzed using an InCell Analyzer 2200 system (Molecular Devices, San Jose, CA, USA) to assess oligonucleotide uptake in CD4^+^ lymphocytes. Nuclei were stained with DAPI (blue), CD4 is shown in green (FITC), and oligonucleotides in red (Cy5). Rows correspond to CTLA-4 ASO (top), scrambled oligonucleotide (middle), and PBS control (bottom). Columns show transmitted-light images (left), DAPI/FITC merge (middle), and DAPI/FITC/Cy5 merge (right). Red fluorescence was detected in cells treated with Cy5-labeled oligonucleotides, confirming cellular uptake, whereas no Cy5 signal was observed in PBS-treated cells. (**B**) Cell viability analysis. Splenocytes were treated for 48 h with rSsEno, CTLA-4 ASO, scrambled oligonucleotide, or their combinations. Apoptosis and necrosis were assessed by Annexin V–FITC/PI staining and flow cytometry. The percentages of viable (PI^−^ Annexin V^−^), early apoptotic (PI^−^ Annexin V^+^), late apoptotic (PI^+^ Annexin V^+^), and necrotic (PI^+^ Annexin V^−^) cells are shown. Cell viability remained above 90% across all groups. Heat-treated splenocytes (56 °C for 30 min) were included as a positive control.

As shown in Figure 2A, splenic CD4^+^ lymphocytes efficiently internalized both the Cy5-labeled CTLA-4 ASO and the scrambled oligonucleotide, as evidenced by detectable intracellular red fluorescence. In contrast, no Cy5 signal was observed in PBS-treated cells. The fluorescence pattern indicates successful cellular uptake of both oligonucleotides without apparent differences in distribution between ASO and scrambled control. Cell viability analysis is presented in Figure 2B. Across all experimental conditions, including rSsEno, CTLA-4 ASO, scrambled oligonucleotide, and their combinations, the percentage of viable cells remained above 90%. Only minimal levels of early apoptosis, late apoptosis, or necrosis were detected, with no significant differences among groups. These results indicate that the treatments did not induce relevant cytotoxic effects under the experimental conditions tested.

### 3.3. CTLA-4 Expression Kinetics and In Vitro Inhibition by CTLA-4 ASO

**Figure 3:**
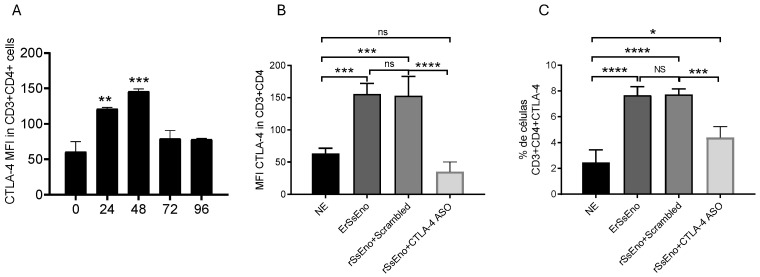
Kinetics of CTLA-4 expression and in vitro inhibitory activity of CTLA-4 ASO. (**A**) CTLA-4 expression kinetics in CD3^+^CD4^+^ T cells following antigen-specific stimulation. BALB/c mice were immunized with rSsEno formulated with Gel 01, and splenocytes were harvested and restimulated in vitro. CTLA-4 mean fluorescence intensity (MFI) increased over time, reaching a peak at 48 h, which was selected for subsequent inhibition assays. (**B**,**C**) In vitro evaluation of CTLA-4 ASO-mediated activity at 48 h. Splenocytes were cultured with medium alone (NE), rSsEno, rSsEno + scrambled or rSsEno plus CTLA-4 ASO. Antigen stimulation increased CTLA-4 expression in CD3^+^CD4^+^ T cells, whereas CTLA-4 ASO treatment reduced CTLA-4 levels compared to antigen-stimulated controls. Data are presented as mean ± SD (n = 3). Statistical analysis was performed using one-way ANOVA followed by Tukey’s multiple comparison test. Statistical significance is indicated as follows: * *p* < 0.05, ** *p* < 0.01, *** *p* < 0.001, **** *p* < 0.0001; ns, not significant.

CTLA-4 expression kinetics in CD3^+^CD4^+^ T cells following rSsEno stimulation are shown in Figure 3A. CTLA-4 mean fluorescence intensity (MFI) increased significantly at 24 h and reached a maximum at 48 h compared to baseline (0 h), followed by a decline at 72 and 96 h. These data established 48 h as the peak time point for CTLA-4 expression. Stimulation with rSsEno significantly increased both CTLA-4 MFI (Figure 3B) and the percentage of CTLA-4^+^ CD3^+^CD4^+^ T cells (Figure 3C) compared to non-stimulated controls. The scrambled oligonucleotide did not significantly alter CTLA-4 expression relative to rSsEno alone. In contrast, treatment with CTLA-4 ASO significantly reduced CTLA-4 MFI and the proportion of CTLA-4^+^ CD4^+^ T cells, supporting effective in vitro inhibition of CTLA-4 expression.

### 3.4. Immunogenicity Study

**Figure 4:**
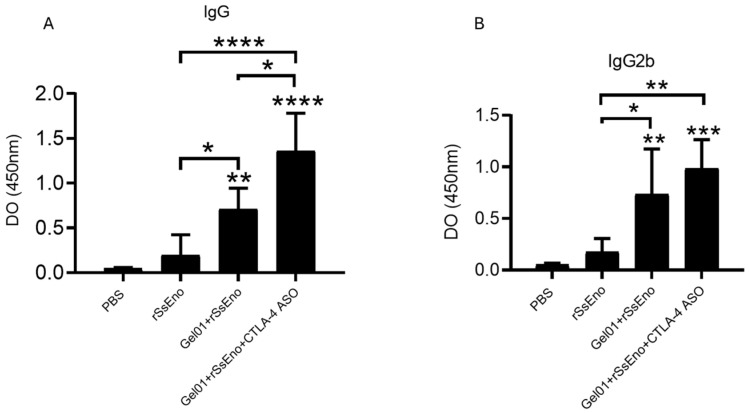
Antigen-specific IgG and IgG2b antibody responses induced by vaccination. Serum levels of rSsEno-specific IgG (**A**) and IgG2b (**B**) were measured by ELISA in mice (n = 5 per group) immunized with PBS, rSsEno, Gel 01 + rSsEno, or Gel 01 + rSsEno + CTLA-4 ASO. Mice immunized with rSsEno showed significantly higher antibody levels compared to PBS controls. The addition of Gel 01 further increased IgG and IgG2b responses. The formulation containing Gel 01 + rSsEno + CTLA-4 ASO induced higher antibody responses, exceeding those observed with rSsEno alone or Gel 01 + rSsEno. Data are presented as mean ± SD. Statistical analysis was performed using one-way ANOVA followed by Tukey’s multiple comparison test. Statistical significance is indicated as follows: * *p* < 0.05, ** *p* < 0.01, *** *p* < 0.001, **** *p* < 0.0001.

Antigen-specific humoral responses were evaluated by ELISA, as shown in Figure 4. Mice immunized with rSsEno alone developed detectable levels of anti-rSsEno IgG and IgG2b antibodies compared to PBS controls. The formulation containing Gel 01 + rSsEno further increased antibody titers, in line with the expected adjuvant effect of Gel 01. Importantly, the group immunized with Gel 01 + rSsEno + CTLA-4 ASO exhibited a significant enhancement in antibody production compared to rSsEno alone and to the Gel 01 + rSsEno group. Both total IgG and IgG2b levels were elevated in the ASO-treated group, showing that the ASO-containing formulation was associated with enhanced antigen-specific humoral responses. These results suggest a potential immunomodulatory effect that may contribute to enhanced vaccine-induced humoral responses.

**Figure 5:**
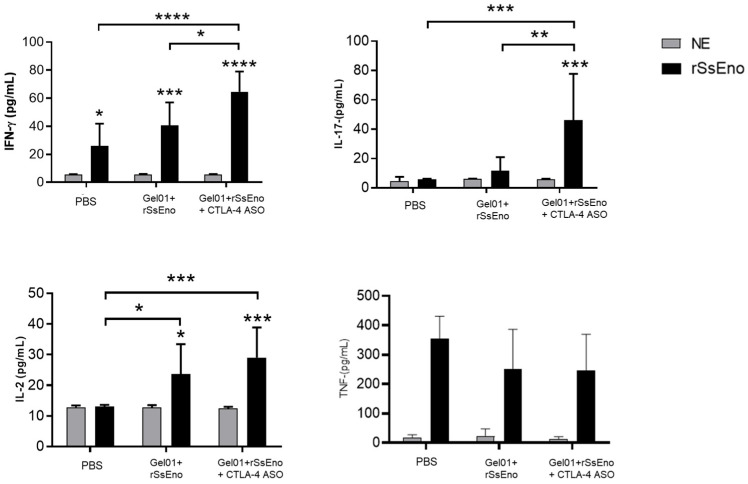
Cytokine profile induced by vaccination with Gel 01 + rSsEno + CTLA-4 ASO. Cytokine levels were measured in supernatants of splenocytes from immunized mice (n = 5 per group) after ex vivo stimulation with rSsEno. The formulation containing Gel 01 + rSsEno + CTLA-4 ASO was associated with increased cytokine responses, particularly IFN-γ and IL-17, compared to groups without ASO. Data are presented as mean ± SD. Statistical analysis was performed using one-way ANOVA followed by Tukey’s multiple comparison test. Statistical significance is indicated as follows: * *p* < 0.05, ** *p* < 0.01, *** *p* < 0.001, **** *p* < 0.0001.

As shown in Figure 5, splenocytes from mice immunized with Gel 01 + rSsEno + CTLA-4 ASO exhibited a significantly higher cytokine response upon ex vivo stimulation with rSsEno compared with groups without ASO. Notably, IFN-γ and IL-17 production significantly increased in the ASO-treated group, consistent with increased Th1- and Th17-associated responses. Although IL-2 levels showed an upward trend, the increase did not reach statistical significance. In contrast, TNF-α production remained unchanged, suggesting that the CTLA-4 ASO-containing formulation is associated with modulation of specific T-cell-associated cytokine responses rather than a generalized pro-inflammatory effect.

## 4. Discussion

Synthetic oligonucleotides, including ASOs, have emerged as versatile tools for modulating immunoregulatory pathways and have been explored as molecular adjuvants to enhance antigen-specific immune responses [26]. Multiple ASOs have been developed to modulate immunoregulatory elements, including cytokines [27,28], immune checkpoint molecules [11,29], transcription factors [14,15], and metabolic immunoregulatory enzymes such as indoleamine 2,3-dioxygenase (IDO) [30]. Across these applications, ASO-based interventions have consistently demonstrated a significant capacity to enhance vaccine-induced immunogenicity.

In this study, we employed a CTLA-4 ASO designed to bind within Exon 1 of the transcript. The exon-localized positioning is mechanistically relevant because this region is present in both precursor and mature mRNA. This enables transcript suppression through established antisense mechanisms. RNase H-competent ASOs induce cleavage and degradation of the target RNA upon duplex formation [31,32]. Additionally, binding within exonic sequences may interfere with splice-site recognition or exonic splicing enhancer elements through steric hindrance, further compromising mRNA maturation [31].

Efficient intracellular delivery is a prerequisite for ASO activity, as hybridization to target RNA occurs predominantly within the cytoplasm and/or nucleus. The detectable Cy5 signal in splenic CD4^+^ lymphocytes demonstrates that both the CTLA-4 ASO and the scrambled oligonucleotide are readily internalized under the experimental conditions used. The absence of Cy5 fluorescence in PBS-treated cells confirms the specificity of the signal. Notably, the similar intracellular distribution of the ASO and scrambled control suggests sequence-independent uptake, likely mediated by non-specific endocytic pathways [31,32,33,34]. The ability of primary lymphocytes to internalize phosphorothioate-modified ASO without transfection reagents has been previously described and is attributed to interactions with cell-surface proteins that facilitate adsorptive or receptor-mediated endocytosis [34,35].

Equally important, cell viability remained above 90% across all treatment groups, with minimal induction of early apoptosis, late apoptosis, or necrosis. These findings indicate that neither the CTLA-4 ASO nor the scrambled control exerted detectable cytotoxic effects at the concentration and exposure time tested. The lack of significant differences between treated and control groups supports the notion that the observed biological effects in subsequent assays are unlikely to be secondary to non-specific toxicity. This is in line with the established safety profile of second-generation chemically modified ASOs, which are generally well tolerated in primary immune cells at micromolar concentrations [36,37,38].

The observed kinetics of CTLA-4 expression after rSsEno stimulation reflects its role as an activation-induced inhibitory receptor. CTLA-4 is upregulated following TCR engagement and functions as a critical negative regulator of T cell responses by competing with CD28 for binding to CD80/CD86 on antigen-presenting cells, thereby limiting costimulatory signaling [2]. In addition to ligand competition, CTLA-4 can actively remove CD80/CD86 from the surface of antigen-presenting cells through trans-endocytosis, further attenuating T cell activation [39]. The significant increase in CTLA-4 expression at 48 h following antigen-specific stimulation reflects engagement of this physiological negative feedback loop. Importantly, treatment with the CTLA-4 ASO significantly reduced both CTLA-4 MFI and the proportion of CTLA-4^+^ CD4^+^ T cells at the peak expression time point, suggesting an effective gene silencing under activation conditions. Given that blockade or downregulation of CTLA-4 enhances CD28-mediated coestimulation and promotes effector T cell expansion [40], our findings support a potential contribution of checkpoint modulation to potentiate antigen-driven immune responses.

A potential concern when using synthetic oligonucleotides is their ability, under certain conditions, to induce innate immune activation, particularly through receptors such as TLR9. However, several features of the ASO used in this study argue against significant non-specific immunostimulatory effects. The sequence does not contain CpG dinucleotide motifs, which are the primary determinants of TLR9 activation, as this receptor preferentially recognizes unmethylated CpG sequences within specific contexts [41]. In addition, the ASO incorporates 2′-O-methyl modifications, which are known to reduce innate immune recognition and can even suppress Toll-like receptor-mediated responses [42,43]. Activation of cytosolic nucleic acid sensors such as RIG-I, MDA5, or the cGAS–STING pathway is also unlikely. These pathways typically require double-stranded or structurally distinct nucleic acids, whereas the ASO used here is short, single-stranded, and chemically modified [44,45,46]. Supporting this interpretation, TNF-α production was not increased in our study (Figure 5), indicating no detectable non-specific innate immune activation within the limits of the experimental design. Similarly, scrambled oligonucleotides have been used in previous vivo studies and in our own pilot experiments without evidence of non-specific immune activation [14,15]. Although these observations argue against a major role for direct innate immune activation, such effects cannot be fully excluded without sequence-independent controls. Accordingly, rigorous validation of sequence specificity will require future studies incorporating appropriate controls, including scrambled oligonucleotides, with a particular focus on in vivo validation.

The dose used in this study (5 µg/mouse) was selected based on previous experimental work in which ASOs were evaluated across a range of doses (1–8 µg/mouse), with no substantial differences in biological responses observed within this interval, indicating a relatively broad effective window under similar conditions [14]. Accordingly, an intermediate dose of 5 µg/mouse was chosen as a representative and reproducible condition, consistent with previously reported ASO-based approaches [6]. Under these conditions, incorporation of the CTLA-4-targeting ASO into an adjuvanted rSsEno formulation was associated with antigen-specific antibody responses and selectively increased recall IFN-γ and IL-17 production. These immunopotentiating effects were achieved while maintaining low in vitro cytotoxicity and suggesting effective inhibition of CTLA-4 expression in antigen-stimulated CD4^+^ T cells. Together, these findings are in line with the concept that attenuation of inhibitory immune checkpoints during antigen priming can enhance antigen-specific immune responses without requiring permanent immune reprogramming [5].

The antibody enhancement observed in our study confirm previous findings showing the immunogenicity and protective relevance of rSsEno as a vaccine antigen. Portuondo and colleagues showed that rSsEno formulations induce robust antigen-specific IgG responses, including IgG2b, which correlate with improved fungal clearance and protection in murine sporotrichosis models [25]. Moreover, rSsEno-based vaccines have been associated with balanced Th1/Th17 responses, both of which are critical for antifungal immunity [20,25,47,48]. In this context, the increased antibody titers and the mixed Th1/Th17 cytokine profile observed with the CTLA-4 ASO-containing formulation likely reflect an amplification of previously described protective pathways rather than the induction of a qualitatively distinct immune response. However, additional mechanisms cannot be excluded, and future studies may reveal further immunomodulatory effects.

This study has several limitations inherent to its proof-of-concept design, which aimed to evaluate the feasibility of modulating CTLA-4 expression as an immunomodulatory strategy. A scrambled ASO control was not included in the in vivo experiments; however, the scrambled oligonucleotide used here lacks structural features typically associated with innate immune activation and has not shown immunostimulatory effects in previous assays, making non-specific activation unlikely. In addition, CTLA-4 modulation was evaluated primarily in CD3^+^CD4^+^ T cells, without comprehensive analysis of other relevant subsets such as CD8^+^ or regulatory T cells. In vivo pharmacokinetics and biodistribution were not assessed, and a single ASO dose was used without formal dose–response analysis. Moreover, although increased antibody responses and cytokine production were observed, no infection challenge experiments were performed, precluding conclusions regarding protective efficacy. Future studies will address these aspects to further define the mechanism and translational potential of this approach.

## 5. Conclusions

Overall, our data support checkpoint targeting as a proof-of-concept immunomodulatory strategy. Administration of CTLA-4 ASO as part of the vaccine formulation was associated with increased antigen-specific humoral responses and a mixed Th1/Th17 recall profile, both of which have been linked to antifungal immunity in experimental models. These observations are consistent with a potential reduction in CTLA-4-mediated inhibitory signaling during immune priming and are in line with previous evidence that CTLA-4 targeting can enhance antigen-specific immune responses. Importantly, these findings should be interpreted with caution, as the current study does not establish a direct causal relationship between CTLA-4 silencing and protective immunity. Future studies will be required to define optimal conditions, confirm sequence specificity, elucidate underlying mechanisms, and evaluate the translational potential of this strategy in antigen-specific immunization settings.

## Figures and Tables

**Figure A2:**
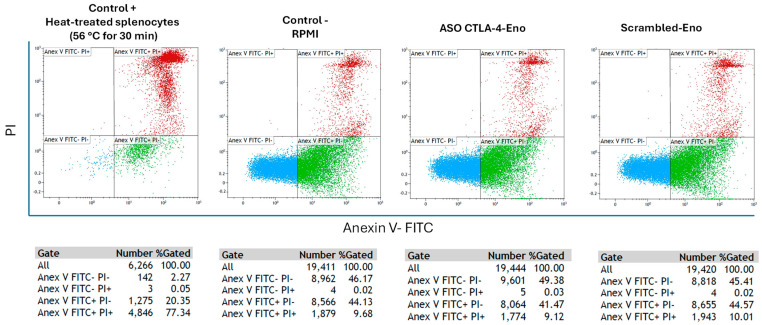
Representative Annexin V/PI Flow Cytometry Profiles of Apoptosis and Necrosis in Mouse Splenocytes.

## Data Availability

The data presented in this study related to the undergraduate thesis are not publicly available due to institutional restrictions. The corresponding author may provide the data upon reasonable request and with appropriate authorization.

## Abbreviations

The following abbreviations are used in this manuscript:

ANOVA	Analysis of Variance
APC	Allophycocyanin
ASO	Antisense Oligonucleotide
BSA	Bovine Serum Albumin
CTLA-4	Cytotoxic T-Lymphocyte-associated Protein 4
CBA	Cytometric Bead Array
ELISA	Enzyme-Linked Immunosorbent Assay
FBS	Fetal Bovine Serum
FITC	Fluorescein Isothiocyanate
FOXP3	Forkhead Box P3
FSC	Forward Scatter
HRP	Horseradish Peroxidase
IDO	Indoleamine 2,3-Dioxygenase
IFN-γ	Interferon Gamma
IgG	Immunoglobulin G
IL	Interleukin
mAb	Monoclonal Antibody
MFI	Mean Fluorescence Intensity
NCBI	National Center for Biotechnology Information
OD	Optical Density
ON	Oligonucleotide
PBS	Phosphate-Buffered Saline
PBS-T	Phosphate-Buffered Saline with Tween 20
PE	Phycoerythrin
PI	Propidium Iodide
rSsEno	Recombinant *Sporothrix* sp. Enolase
RNAi	RNA Interference
RPMI	Roswell Park Memorial Institute Medium
SD	Standard Deviation
SSC	Side Scatter
TCR	T-Cell Receptor
TGF-β	Transforming Growth Factor Beta
Th	T Helper
TLR9	Toll-Like Receptor 9
TNF-α	Tumor Necrosis Factor Alpha
TMB	3,3′,5,5′-Tetramethylbenzidine
Treg	Regulatory T Cell
